# Cancer Survival and Excess Mortality Estimates among Adolescents and Young Adults in Western Australia, 1982–2004: A Population-Based Study

**DOI:** 10.1371/journal.pone.0055630

**Published:** 2013-02-06

**Authors:** Fatima A. Haggar, Gavin Pereira, David D. Preen, C. D’Arcy J Holman, Kristjana Einarsdottir

**Affiliations:** 1 School of Population Health, Centre for Health Services Research, The University of Western Australia, Crawley, Australia; 2 The Ottawa Hospital Research Institute, The University of Ottawa, Ottawa, Ontario, Canada; 3 Telethon Institute for Child Health Research, Centre for Child Health Research, University of Western Australia, Subiaco, Australia; 4 Yale Center for Perinatal, Pediatric, and Environmental Epidemiology, School of Public Health, Yale University, New Haven, Connecticut, United States of America; College of Pharmacy, University of Florida, United States of America

## Abstract

**Background:**

Data are limited on cancer outcomes in adolescents and young adults.

**Methods:**

Based on data from the Western Australian Data Linkage System, this study modelled survival and excess mortality in all adolescents and young adults aged 15–39 years in Western Australia who had a diagnosis of cancer in the period 1982–2004. Relative survival and excess all-cause mortality for all cancers combined and for principal tumour subgroups were estimated, using the Ederer II method and generalised linear Poisson modelling, respectively.

**Results:**

A cancer diagnosis in adolescents and young adults conferred substantial survival decrement. However, overall outcomes improved over calendar period (excess mortality hazard ratio [HR], latest versus earliest diagnostic period: 0.52, trend p<0.0001). Case fatality varied according to age group (HR, oldest versus youngest: 1.38, trend p<0.0001), sex (HR, female versus male: 0.66, 95% confidence interval [CI] 0.62–0.71), ethnicity (HR, Aboriginal versus others: 1.47, CI 1.23–1.76), geographical area (HR, rural/remote versus urban: 1.13, CI 1.04–1.23) and residential socioeconomic status (HR, lowest versus highest quartile: 1.14, trend p<0.05). Tumour subgroups differed substantially in frequency according to age group and sex, and were critical outcome determinants.

**Conclusions:**

Marked progressive calendar-time improvement in overall outcomes was evident. Further research is required to disentangle the contributions of tumour biology and health service factors to outcome disparities between ethno-demographic, geographic and socioeconomic subgroups of adolescents and young adults with cancer.

## Introduction

Cancers in adolescents and young adults (AYAs), commonly defined as persons aged 15–39 years [1], have a distinctive spectrum of pathology, different from that evident in children (<14 years) and older adults. These malignancies comprise a mixture of the non-epithelial cancers that commonly occur during childhood and those of epithelial origin that account for most cancers in older adults [1], [2]. Furthermore, AYAs are not homogeneous as a group, with substantial biological and psychosocial differences pertinent to cancer outcomes evident across the broad AYA age range.

Compared with children and older adults, patients in the AYAs age group have reportedly experienced little or no improvement in cancer survival in more than two decades, [1]. Possible contributory factors for the deficit in survival improvement include: delays in seeking treatment, delays in recognition of malignancy by physicians, lack of participation in clinical trials and low uptake of private health insurance [3]–[5]. Fortunately, recent international advocacy has led to better dissemination of research findings and greater professional awareness concerning cancer in the AYA population [6]. However, few empirical studies to date have focused specifically on relative survival and excess mortality experienced by AYA cancer patients. Such long-term survival estimates are not only necessary for planning the healthcare response to the cancer burden in this age-group, but also for national and international comparisons between jurisdictions with different environments and/or health care systems. Further, they can serve as a foundation for appropriate surveillance, including the management of long-term sequelae, such as late recurrence, second primary cancers and other delayed disease-related and iatrogenic complications [7]. In this paper, we estimate relative survival ratios (RSR) and model excess mortality from malignancies in AYAs, highlighting differences in outcomes according to gender, age, socio-economic status, geographic location and calendar period of diagnosis.

## Methods

### Population and Data Source

Data on all individuals first diagnosed with invasive cancer or lymphohaematopietic malignancy at 15–39 years of age during the period 1982–2004 were extracted from the Western Australian Data Linkage System (WADLS). Notification of all cancer diagnoses has been a statutory requirement for all public and private hospitals and pathology services in WA since 1981 [8], and so the data reported here are considered to represent all eligible individuals. Information obtained included demographic data (date of birth, sex, Aboriginality, area/geozone of residence), tumour-specific details (date and basis/modality of original diagnosis, anatomical site, histology, behaviour, grade, date of diagnosis and characteristics of subsequent malignancies) and vital status. Active follow-up to December 31 2007 was performed through linkage of data provided by the WA Cancer Registry (WACR), the WA Mortality Register and the Australian National Death Index. Malignancies were classified according to histological origin, as described in the third edition of the *International Classification of Diseases for Oncology* (ICD-O) [9], and further characterized according to cancer subtypes based on the AYA classification scheme published by The US Surveillance, Epidemiology, and End Results Program (SEER) [10]. The SEER subgroups were developed to better define the major cancer sites that affect individuals aged 15–39 years [11].

### Measurement of Co-morbidity, Area-based SES and Residential Remoteness

Routine and unprecedentedly accurate geocoding of health records is a unique feature of the WADLS. This enables anonymised identification of the residential location of patients (including categorisation of remoteness), from which socio-economic status (SES) can be inferred. SES was measured using the Index of Relative Socio-economic Disadvantage (IRSD), which is based on Australian census data elements, including the prevalence of low income, low educational attainment, unemployment, rented dwellings, one-parent families, and other measures of social disadvantage such as prevalence of poor English language fluency [12]. Subjects were classified into four SES groups, based on WA population quartiles (1^st^-25^th^ centiles, most disadvantaged; 76^th^-100^th^ centiles, least disadvantaged). The degree of residential remoteness was based on the Australian Bureau of Statistics (ABS) Accessibility/Remoteness Index of Australia (ARIA) codes, which use distances to population centres as the basis for quantifying service access [13]. For the purpose of this study, ARIA categories were collapsed into two groups; urban (major cities) and rural & remote (inner regional, outer regional, remote, and very remote), due to the smaller numbers of patients in regional and remote areas. The Charlson Co-morbidity Index (CCI), a weighted composite score of 17 different chronic conditions, was used to adjust for the effects of co-morbidity [14].

### Analysis

Relative survival ratio (RSR) was used to estimate disease-specific survival. RSR is defined as the ratio of the observed survival in the diseased individuals under study to the expected survival of the underlying general population in WA according to sex, age and calendar year of death. WA population estimates were supplied by the ABS [15]. The major advantage of analysis based on relative survival is that information on cause of death is not required [16]. It provides a measure of excess mortality among cancer patients, irrespective of whether or not deaths have been medically certified as directly or indirectly cancer-induced/related [16], [17]. The Ederer II method, described elsewhere [18], was used to calculate expected survival. Cancer cases classified in the absence of histological confirmation, on the basis of death certificate only (DCO) or other modality of diagnosis (e.g. hospital record only), were excluded from the analysis. Generalised linear models with Poisson error structures were used to model the excess all-cause mortality associated with a diagnosis of cancer (up to 10 years after diagnosis) for all cancers combined and within each principal diagnostic subgroup, including the effects of age, sex, Aboriginality, co-morbidity, calendar period of diagnosis, length of follow-up, SES, and area of residence. Poisson modelling was not applied to subgroup analysis of bone sarcomas, due to small sample size. Analyses were performed using SAS 9.2 [19].

### Ethics

Ethics approval for this study was obtained from the University of Western Australia Research Ethics Committee (reference number: RA/4/1/2228). The Ethics Committee waived the need for participants’ written informed consent as this was a minimal-risk retrospective study, exclusively based on data extraction from administrative databases, and it would not be feasible to get patients’ consent for access to all charts. According to Australia human research law, informed consent can be waived in cases for which recording informed consent is not possible, provided that a justification is registered and an Ethics Committee gives approval. The data were analysed anonymously.

## Results

### Description of Cohort

**Figure 1:**
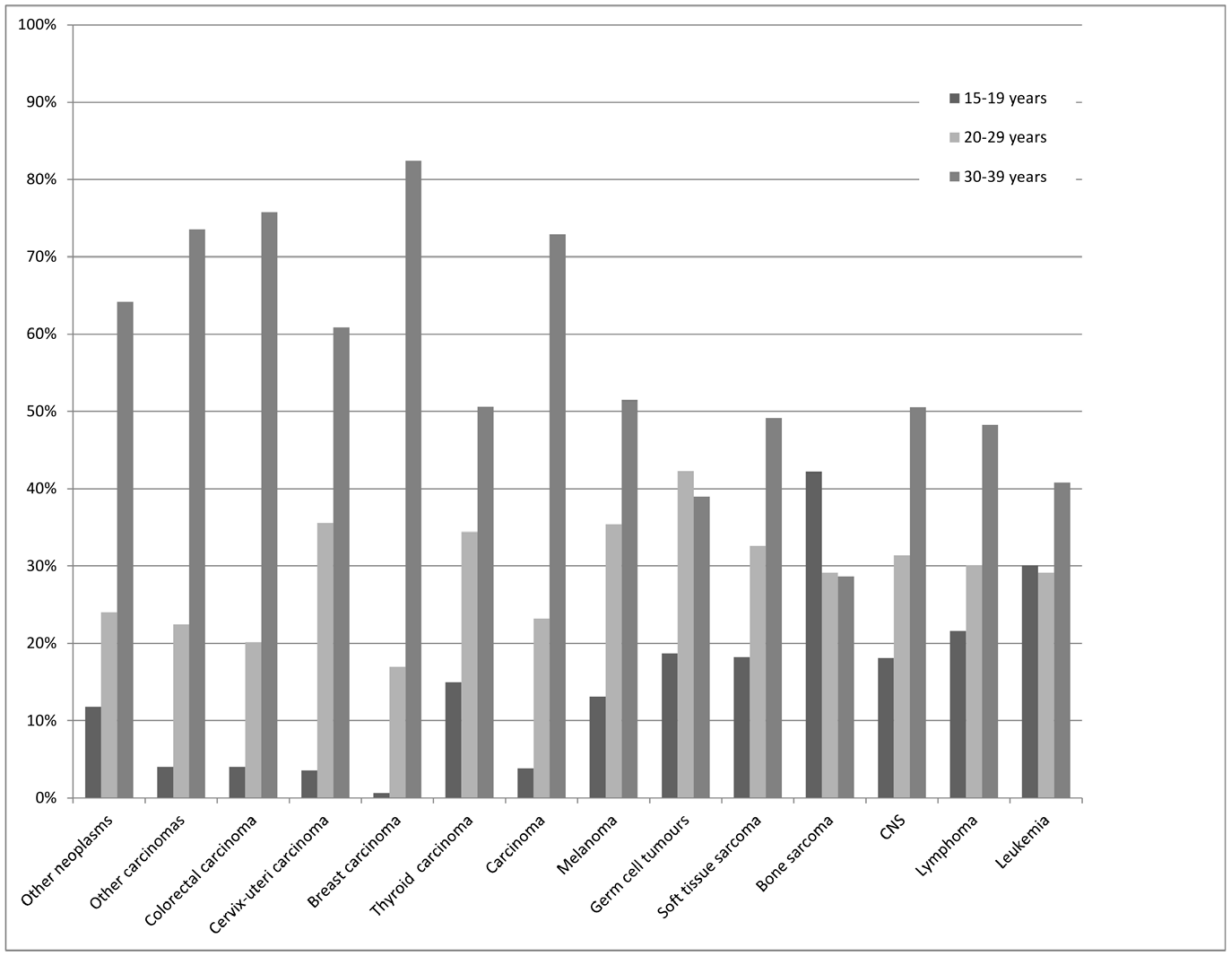
Distribution of each diagnostic groups and selected carcinomas displayed by age group.

There were 10,266 incident cases of malignant neoplasms reported in WA among AYAs aged 15–39 years in 1982–2004. Based on all-cause mortality, the proportions of AYA cancer patients alive at 1, 5 and 10 years post-diagnosis were 91.8%, 75.6% and 49.8%, respectively. The median follow-up time was 8.2 years (interquartile range: 12 years). Figure 1 displays the distribution of each diagnostic subgroup and selected carcinomas by age group and sex, respectively. More than 98% of the cancer registrations were histologically confirmed cases and 0.1% were DCO diagnoses. The total of number of cases included in the study, and the proportions of DCO diagnoses and cases verified histologically are shown for each diagnostic subgroup in Table 1.

**Table 1:** Total of number of cases, sex ratio and proportion of cases verified microscopically.

	No. of cases, (%)		Sex ratio(M:F)	MicroscopicVerification (%)	DCO(%)
**All Cancers**	10266	(100)	0.7	98.6	0.06
**Leukaemias**	384	(3.7)	1.5	96.4	0
**Lymphoma**	806	(7.9)	1.4	97.9	0.1
**CNS tumours**	350	(3.4)	1.3	87.7	0
**Bone sarcoma**	148	(1.4)	1.4	98.8	0
**Soft tissue sarcoma**	254	(2.4)	1.5	95.6	0.28
**Germ cell tumours**	746	(7.2)	10.0	99.1	0
**Melanoma**	3127	(30.1	0.9	99.3	0.02
**Carcinoma**	4291	(41.8)	0.4	99.2	0.03
Thyroid	528	(5.1)	0.3	99.7	0
Breast	307	(12.7)	–	99.5	0
Cervix uteri	699	(6.8)	–	99.4	0
Colorectum	357	(3.5)	1.1	99.4	0
Other	610	(5.9)	0.7	98.5	0.1
**Other Neoplasms**	160	(1.6)	0.7	79.5	1.6

### Relative Survival: Overall and within Diagnostic Subgroups

Table 2 shows 5-year and 10-year RSR for the most recent study period, 2000–2004, by diagnostic subgroup. Overall 5-year RSR for AYAs diagnosed with any cancer was 0.84 (95% CI 0.82–0.86) in males and 0.86 (0.85–0.88) in females. Favourable survival prospects were observed for AYAs with melanoma (males: RSR = 0.96, 0.92–0.96; females: RSR = 0.98, 0.96–0.99) and for males with germ cell tumours (RSR = 1.00, 0.97–1.00). Among females with germ cell tumours, average 5-year RSR exceeded 0.90 only in the youngest females (15–19 years). RSR for AYAs with carcinomas was better in females compared with males (0.71, 0.67–0.76 versus 0.85, 0.83–0.87). AYAs diagnosed with lymphoma had encouraging prognoses with an overall 5-year RSR of approximately 0.85. In contrast, diagnoses of leukaemias and central nervous system (CNS) malignancies carried poor prognoses, with relatively low 5-year RSRs of approximately 0.60 and 0.50, respectively.

**Table 2:** 5- and 10-year relative survival ratio for the most recent diagnosis period, 2000–2004.^a^

		Males		Females	
		5-yr RSR (95% CI)	10-yr RSR (95% CI)	5-yr RSR (95% CI)	10-yr RSR (95% CI)
**All Cancers**	15–19	0.85 (0.79, 0.90)	0.80 (0.73, 0.85)	0.92 (0.85, 0.95)	0.81 (0.71, 0.88)
	20–29	0.89 (0.86, 0.92)	0.88 (0.84, 0.90)	0.87 (0.83, 0.90)	0.79 (0.75, 0.83)
	30–39	0.81 (0.78, 0.83)	0.75 (0.71, 0.78)	0.85 (0.83, 0.87)	0.81 (0.79, 0.82)
	15–39	0.84 (0.82, 0.86)	0.78 (0.75, 0.81)	0.86 (0.85, 0.88)	0.81 (0.78, 0.83)
**Leukaemias**	15–19	0.64 (0.52, 0.86)	0.53 (0.30, 0.72)	0.62 (0.49, 0.85)	0.45 (0.25, 0.67)
	20–29	0.73 (0.57, 0.83)	0.57 (0.33, 0.72)	0.69 (0.53, 0.94)	0.62 (0.38, 0.71)
	30–39	0.57 (0.44, 0.82)	0.54 (0.34, 0.73)	0.54 (0.34, 0.83)	0.44 (0.28, 0.63)
	15–39	0.62 (0.46, 0.75)	0.55 (0.35, 0.67)	0.61 (0.33, 0.87)	0.46 (0.36, 0.54)
**Lymphoma**	15–19	0.92 (0.73, 0.99)	0.93 (0.72, 0.98)	0.97 (0.94, 0.99)	0.94 (0.65, 0.99)
	20–29	0.89 (0.73, 0.96)	0.94 (0.82, 0.98)	0.85 (0.61, 0.95)	0.83 (0.63, 0.93)
	30–39	0.81 (0.67, 0.88)	0.71 (0.58, 0.82)	0.87 (0.72, 0.94)	0.83 (0.69, 0.91)
	15–39	0.85 (0.77, 0.90)	0.81 (0.71, 0.88)	0.84 (0.74, 0.91)	0.85 (0.74, 0.92)
**Melanoma**	15–19	0.62 (0.34, 0.89)	0.47 (0.15, 0.85)	0.57 (0.23, 0.88)	0.46 (0.11, 0.80)
	20–29	0.66 (0.38, 0.83)	0.51 (0.29, 0.69)	0.52 (0.26, 0.73)	0.33 (0.15, 0.50)
	30–39	0.56 (0.35, 0.78)	0.35 (0.19, 0.52)	0.61 (0.26, 0.84)	0.45 (0.24, 0.65)
	15–39	0.55 (0.41, 0.71)	0.42 (0.25, 0.57)	0.52 (0.36, 0.65)	0.43 (0.22, 0.63)
**CNS**	15–19	0.76 (0.13, 0.97)	0.67 (0.20, 0.91)	0.97 (0.94, 1.00)	0.84 (0.27, 0.98)
	20–29	0.87 (0.57, 0.97)	0.66 (0.38, 0.83)	0.99 (0.89, 1.00)	0.72 (0.26, 0.93)
	30–39	0.87 (0.63, 0.96)	0.65 (0.39, 0.82)	0.76 (0.51, 0.90)	0.53 (0.20, 0.78)
	15–39	0.83 (0.67, 0.92)	0.65 (0.43, 0.81)	0.81 (0.64, 0.91)	0.70 (0.38, 0.88)
**Soft tissue**	15–19	0.95 (0.79, 0.99)	0.94 (0.74, 0.99)	0.92 (0.72, 0.98)	0.72 (0.41, 0.89)
	20–29	0.99 (0.88, 1.00)	0.98 (0.92, 1.00)	0.85 (0.60, 0.97)	0.80 (0.20, 0.97)
	30–39	0.91 (0.80, 0.96)	1.00 (0.94, 1.00)	0.84 (0.27, 0.98)	0.84 (0.27, 0.97)
	15–39	1.00 (0.97, 1.00)	0.94 (0.87, 0.97)	0.87 (0.57, 0.97)	0.73 (0.46, 0.88)
**Germ Cell**	15–19	0.95 (0.81, 0.99)	0.95 (0.84, 0.98)	0.97 (0.93, 0.99)	0.93 (0.69, 0.99)
	20–29	0.97 (0.93, 0.99)	0.97 (0.93, 0.99)	0.98 (0.95, 1.00)	0.98 (0.95, 1.00)
	30–39	0.96 (0.90, 0.97)	0.93 (0.89, 0.96)	0.98 (0.95, 0.99)	0.98 (0.95, 1.00)
	15–39	0.96 (0.92, 0.96)	0.95 (0.91, 0.97)	0.98 (0.96, 0.99)	0.97 (0.94, 0.99)
	15–19	0.88 (0.60, 0.97)	0.65 (0.38, 0.83)	0.97 (0.79, 1.00)	0.75 (0.48, 0.90)
**Carcinomas**	20–29	0.80 (0.70, 0.87)	0.80 (0.70, 0.88)	0.86 (0.81, 0.90)	0.72 (0.64, 0.78)
	30–39	0.68 (0.62, 0.73)	0.60 (0.54, 0.66)	0.84 (0.81, 0.87)	0.78 (0.75, 0.81)
	15–39	0.71 (0.67, 0.76)	0.62 (0.56, 0.68)	0.85 (0.83, 0.87)	0.76 (0.73, 0.79)

a Bone Sarcoma not included among subgroup analyses; CI: confidence intervals.

### Excess Mortality by Period of Diagnosis and Length of Follow-up

Adjusted excess mortality hazard ratios in AYAs by diagnostic subgroup and calendar period of diagnosis and time since diagnosis are shown in Table 3. With the exception of the CNS and soft tissue sarcoma tumour subtypes, excess mortality from cancer decreased over calendar time (group effect p-value <0.0001). AYA patients diagnosed in the period 2000–2004 with any type of cancer were estimated to have a 52% (HR 0.50, CI 0.454–0.60.58) lower excess mortality compared with those diagnosed in the earliest half-decade (1985–1989).

**Table 3:** Adjusted excess mortality hazard ratios by diagnostic group, calendar period of diagnosis and years of follow-up, 1982–2004.a,b

	Calendar period of diagnosisReference: 1985–1989				Follow-up timeReference: Year 1				
	1990–1994	1995–1999	2000–2004		Year 2	Year 3	Year 4	Year 5	
**All cancers**	0.87 (0.44, 0.85)	0.62 (0.52, 0.73)	0.52 (0.45, 0.60)	†	0.64 (0.59, 0.71)	0.38 (0.34, 0.43)	0.31 (0.27, 0.35)	0.22 (0.19, 0.26)	†
**Leukaemia**	1.01 (0.43, 1.37)	0.94 (0.21, 1.67)	0.61 (0.38, 1.00)	†	0.73 (0.54, 0.10)	0.49 (0.34, 0.72)	0.36 (0.23, 0.56)	0.23 (0.13, 0.40)	†
**Lymphoma**	0.87 (0.59, 1.34)	0.62 (0.43, 0.97)	0.48 (0.30, 0.84)	‡	0.59 (0.42, 0.83)	0.35 (0.23, 0.53)	0.12 (0.06, 0.24)	0.22 (0.13, 0.37)	†
**CNS**	0.89 (0.47, 1.57)	1.00 (0.60, 1.71)	1.04 (0.66, 1.74)		0.73 (0.53, 0.99)	0.35 (0.23, 0.54)	0.40 (0.26, 0.61)	0.32 (0.19, 0.52)	†
**Soft tissue**	1.16 (0.70, 2.14)	0.78 (0.29, 1.90)	0.62 (0.32, 1.26)		0.97 (0.62, 1.51)	0.17 (0.07, 0.41)	0.29 (0.14, 0.60)	0.31 (0.15, 0.64)	†
**Germ cell**	0.40 (0.26, 0.85)	0.36 (0.16, 0.95)	0.21 (0.09, 0.39)	‡	0.70 (0.35, 1.39)	0.38 (0.16, 0.90)	0.19 (0.06, 0.64)	0.05 (0.003, 0.82)	†
**Melanoma**	0.91 (0.80, 1.37)	0.37 (0.26, 0.53)	0.31 (0.12, 0.64)	†	0.88 (0.55, 1.41)	0.64 (0.38, 1.09)	0.71 (0.43, 1.19)	0.87 (0.54, 1.41)	
**Carcinoma**	0.88 (0.75, 1.04)	0.78 (0.66, 0.82)	0.59 (0.50, 0.70)	†	0.65 (0.58, 0.74)	0.43 (0.37, 0.50)	0.35 (0.29, 0.41)	0.23 (0.18, 0.27)	†

a model also adjusted for sex, Aboriginal status, age at diagnosis, years of follow-up; ARIA, IRSD and Charlson Index; †: highly significant group effect (p<0.0001); ‡: significant group effect (0.001<p<0.05).^b^ Poisson model was not applied to bone sarcomas because of instability and lack of convergence in the regression model.

For all cancers combined and all studied cancer subgroups except melanoma, the risk of dying decreased significantly with duration of time after diagnosis. The annual risk of death from melanoma remained stable throughout 5 years of follow-up (Table 3).

### Excess Mortality by Age-group

Considerable differences in the spectrum of cancers experienced by the different age groups were noted (Figure 1). Table 4 shows the risk of death by diagnostic group and age, sex, Aboriginal status, location and social disadvantage. For all AYA cancers combined, excess mortality increased with increasing age at diagnosis. When diagnostic subgroups were analysed separately, younger AYAs (aged 15–19 years) diagnosed with leukaemias had significantly worse survival outcomes compared with older AYAs. In other diagnostic groups (i.e., lymphoma, CNS and carcinomas) for which there were a significant age differential, excess mortality was associated with older age at diagnosis.

**Table 4:** Adjusted excess mortality hazard ratios by diagnostic group and sex, Aboriginal status, age, location and social disadvantage, 1982–2004.^a^

	Sex		Aboriginal		Age at diagnosis, years			Location		Social disadvantage (IRSD)			
	Reference: Male		Reference: No		Reference: 15–19			Reference: Urban		Reference: 4^th^ quartile (least disadvantaged)			
	Female		Yes		20–29	30–39		Rural/remote		1^st^ quartile (most)	2^nd^ quartile	3^rd^ quartile	
**All cancers**	0.66 (0.62, 0.71)	†	1.47 (1.23, 1.76)	†	1.07 (0.76, 1.54)	1.38 (1.21, 1.58)	†	1.13 (1.04, 1.23)	‡	1.14 (1.04, 1.26)	1.03 (0.93, 1.13)	1.01 (0.92, 1.16)	‡
**Leukaemia**	1.26 (1.03, 1.63)	‡	1.25 (0.60, 2.63)		0.98 (0.71, 1.36)	0.74 (0.52, 1.06)	†	0.99 (0.70, 1.39)		1.23 (0.85, 1.76)	1.18 (0.82, 1.71)	1.01 (0.71, 1.44)	‡
**Lymphoma**	0.65 (0.49, 0.87)	‡	1.54 (0.67, 3.56)		1.30 (0.81, 2.09)	2.13 (1.39, 3.25)	†	1.10 (0.80, 1.51)		1.07 (0.74, 1.55)	1.29 (0.91, 1.83)	1.30 (0.91, 1.84)	
**CNS**	0.92 (0.71, 1.19)		0.79 (0.36, 1.73)		1.03 (0.67, 1.57)	1.60 (1.09, 2.35)	‡	1.09 (0.82, 1.46)		1.14 (0.82, 1.59)	1.07 (0.78, 1.49)	0.97 (0.52, 1.11)	
**Soft tissue**	0.54 (0.35, 0.83)	‡	1.28 (0.39, 4.21)		0.94 (0.52, 1.71)	1.13 (0.65, 1.95)		1.21 (0.72, 2.03)		1.47 (0.83, 2.59)	1.41 (0.78, 2.57)	0.92 (0.53, 1.61)	
**Germ cell**	3.71 (2.17, 6.36)	‡	6.69 (2.18, 20.6)	†	0.97 (0.69, 1.80)	1.03 (0.61, 1.89)		1.08 (0.58, 2.01)		0.83 (0.42, 1.65)	1.59 (0.84, 3.07)	1.14 (0.55, 2.34)	
**Melanoma**	0.53 (0.38, 0.74)	†	3.63 (0.50, 26.2)		1.19 (0.67,.10)	1.28 (0.74, 2.21)		1.09 (0.83, 1.43)		1.21 (0.87, 1.69)	1.09 (0.79, 1.50)	1.08 (0.79, 1.47)	
**Carcinoma**	0.51 (0.46, 0.56)	†	1.25 (1.01, 1.54)	‡	1.25 (0.88, 1.76)	1.80 (1.30, 2.50)	†	1.38 (1.24, 1.82)	†	0.99 (0.87, 1.12)	1.07 (0.94, 1.21)	0.98 (0.86, 1.12)	

a model also adjusted for years of follow-up, calendar period and Charlson Index; †: highly significant group effect (p<0.0001); ‡: significant group effect (0.001<p<0.05).

### Excess Mortality by Sex

Survival for all cancers combined was poorer in males compared with females (0.66, 0.62–0.71): females with lymphoma (0.65, 0.49–0.87), soft tissue sarcoma (0.54, 0.35–0.83), melanoma (0.53, 0.38–0.74) and carcinomas (0.51, 0.46–0.56) experienced significantly lower excess mortality compared with males (Table 4). In contrast, AYA females diagnosed with germ cell tumours (3.71, 2.17–6.36) experienced higher excess mortality compared with their male counterparts, and higher excess mortality among females with leukaemias approached statistical significance (1.26, 1.03–1.63).

### Excess Mortality by Aboriginal Status

Non-Aboriginal AYAs comprised the majority (n = 9445, 92%) of cancer cases. After adjusting for sex, age at diagnosis, co-morbidity, locational disadvantage, SES, length of follow-up and year of diagnosis, Aboriginality significantly contributed to the risk of death. Overall, Aboriginal AYAs experienced a higher excess mortality (1.47, 1.23–1.76) compared with non-Aboriginal AYAs. Among the diagnostic subgroups, Aboriginal AYAs diagnosed with carcinomas experienced 25% significantly greater excess mortality (1.25, 1.01–1.54) than their non-Aboriginal counterparts; and those diagnosed with germ cell tumours experienced nearly seven times higher excess mortality (6.7, 2.2–20.6). The results for the other subgroups did not reach statistical significance.

### Excess Mortality by Area of Residence and SES

AYAs living in rural and remote areas had an increased risk of mortality compared with those who lived in urban areas (1.13, 1.04–1.23). However, among the diagnostic subgroups, the risk was only significant for those diagnosed with carcinomas (1.38, 1.24–1.82). A significant gradient of increased mortality with declining SES for all cancers combined was observed (HR, lowest versus highest quartile: 1.14, trend p<0.05). Among the cancer subgroups, the adjusted HRs were only significant for AYAs diagnosed with leukaemias (trend p<0.001). Approximately 65% of Aboriginal AYAs (versus 23% non-Aboriginal) resided in rural and remote areas. Aboriginal AYAs in WA were over-represented in the most socially disadvantaged categories, with less than 5% of Aboriginal AYAs cancer patients (versus 27% non-Aboriginal) in the highest quartile (least disadvantaged group).

## Discussion

This paper reports estimates of long-term relative survival and risk of excess mortality in AYAs diagnosed with cancer in WA, using the current SEER classification for AYAs cancer subgroups. Recently treated AYAs had a significantly lower risk of death than those treated earlier in the study period. Older age at of diagnosis was a predictor of poor prognosis for all cancers combined and for lymphoma, CNS tumours and carcinomas indvidually. In general, female AYAs had better survival outcomes compared with their male counterparts. Aboriginality was identified as a poor prognostic factor, particularly among AYAs diagnosed with germ cells tumours. Our results reinforce the importance of both socio-economic status and area of residence in the survival of AYAs diagnosed with cancers.

Recently diagnosed AYAs (2000–2004) in this population were estimated to have 50% lower excess mortality compared with those diagnosed in 1980s. In particular, survival from leukaemias, lymphomas, germ cell tumours, melanoma and carcinomas has improved markedly over the last few decades. This is likely to reflect the increasing availability of better diagnostic techniques and more effective therapies. Male germ cell tumours and melanomas presented the best prognoses with 5-year RSRs of around 0.95 and 1.00, respectively. In a previous study, significant improvements in outcomes from the treatment of germ cell tumours had occurred mainly before the 1980s, coincident with the widespread introduction of platinum-based treatment regimens [20]. Recent improved survival is likely to reflect the fact that, in most cases, germ cell tumours can be cured by adequate treatment. Improvements in melanoma survival have been attributed mainly to earlier cancer detection and increased awareness among young Australians [21].

Of concern are the poor outcomes associated with CNS tumours in this study. It is difficult to ascertain the reasons for these. Unfortunately, WA Cancer Registry data on CNS tumours do not include important predictors of survival, such as histological categorisation (in a substantial minority of cases), extent of disease, and molecular typing such as *N-Myc* expression. Epidemiological research on CNS malignancies is inherently fraught, given the biological heterogeneity of these tumours and the relative rarity of any specific histologic subtype, as well as the challenges associated with capture of pertinent data [5]. Large Multinational prospective cohort studies, similar to the Childhood Cancer Survivor Study [22], would facilitate understanding of CNS neoplasms and other biologically complex cancer subgroups common among AYA.

The association of older age at diagnosis with poorer long-term survival among AYAs diagnosed with lymphoma, CNS and carcinomas is concordant with previous reports for this age group. By contrast, in the case of leukaemia, survival was worse among younger AYAs, aged 15–29 years compared with those older than 30 years. Past research has shown similar disparities, with children and adolescents diagnosed with leukemia. Unlike children with biologically similar leukaemias, younger AYAs are often administered adult rather than paediatric treatment regimens, which may ultimately be less effective [23], [24]. In multiple studies, there is a consistent, large event-free survival and survival advantage for young adult patients treated on paediatric versus adult protocols [25]. However, these past findings do not explain the results of our study.

Male AYAs diagnosed with germ cell tumours had markedly reduced excess mortality compared with their female counterparts had better survival outcomes than females, whereas males diagnosed with lymphoma, soft tissue sarcoma, melanoma and carcinomas had a worse prognosis. Sex did not significantly affect prognosis in those diagnosed with CNS tumours, which is consistent with previous studies [26]. Previous childhood cancers studies have shown that girls with acute lymphoblastic leukaemia generally have a slightly better prognosis than boys with this disease [27], [28]; possibly due to a different immuno-phenotype distribution [29]. However, the modest sex differential favouring male AYAs in this study is unexplained, and has not been previously reported. Concordant with the current findings, sex has been previously reported to be an independent prognostic factor for both non-Hodgkin’s [30] and Hodgkin’s lymphoma [31], with male AYA patients having poorer outcomes than females. Although female gender has been previously reported to be independently associated with increased survival from melanoma in older subjects, no clear explanation for this difference currently exists [32]. Even less is known about the sex disparities for melanoma survival in AYAs. The finding in our study may be explained by higher attention of young women to their bodies [21] and differing screening practices, even though a possibility of hormonal or other sex-specific factors that play a role in modulating the growth and metastatic potential of melanoma [33] cannot be ruled out. The substantially reduced excess mortality in males with germ cell tumours is likely attributable to a prognostically more favourable spectrum of tumours, notably including testicular germ cell cancers [34]. The converse excess mortality gender disparity in the instance of carcinomas may be attributed to the predominance among female AYAs of treatable sex-specific carcinomas, such as those of the breast and cervix [2].

AYAs with cancer resident in rural and remote areas at the time of diagnosis had an increased risk of mortality compared with their urban-dwelling counterparts. Stratified analyses by diagnostic group indicated a socioeconomic gradient in survival in those diagnosed with carcinomas. The observed survival disparity for carcinoma may reflect restricted access to optimal care and low density of health care facilities in rural and remote Australia. Previous studies in WA have indeed identified a service delivery gap for rural residents diagnosed with carcinomas of the colon and rectum [35], breast [36] and lung [36]. Treatment at private hospitals, which are concentrated in urban areas, has also been identified as an independent predictor of survival for colorectal [35], breast [36] and lung [36] cancers.

Our study is the first to examine socioeconomic impacts on survival in Australian AYAs with cancer, demonstrating higher excess mortality in AYAs living in socio-economically disadvantaged groups, regardless of area of residence. The effects of SES on prognosis for AYAs were significant for all cancers combined. However, when groups were analysed individually, a significant gradient was detected only for leukaemias. A similar pattern for leukaemias has been found in another study [37]. These measured effects of SES on survival may be a proxy for differences in health awareness and behaviours and/or access to health services, resulting in delayed presentations and thereby diagnoses at more advanced stages of disease. Accordingly, individuals with the highest SES have better access to private healthcare insurance [38]. AYAs have comparatively low health insurance coverage, with lack of private insurance shown to be associated with worse survival for of carcinomas (breast, colorectal) that commonly occur in AYAs.

Our study also revealed that young Aboriginal people diagnosed with cancer experienced worse survival outcomes compared with non-Aboriginal AYAs. Notably, Aboriginal AYAs diagnosed with germ cell tumours experienced nearly seven-times greater mortality compared with non-Aboriginal AYAs. Although, there is risk of important residual confounding by location due to our stratified analysis of locational disadvantage (urban vs. rural & remote), the difference in survival by Aboriginality persisted after adjustment for area-based SES and locational disadvantage). This would suggest the possibility of important biological or other unknown factors that contribute to worse survival in Aboriginal males. However, it is difficult from our analysis alone to determine whether differences were due to biology or other possible factors such as differential access to adequate treatment or and health behaviours.

### Strengths and Weaknesses

A major strength of this study is its use of routinely-collected, whole-population data from the WADLS, which has undergone extensive validation, with false-positives and false-negatives of subject identification shown to be <1%. Rigorous characterisation of the cancers and careful follow-up were important features of the WACR. Rigorous procedures are implemented to ensure that cancer ascertainment is as complete as possible; cases are identified from multiple sources. In relation to the main indicator of data quality, namely, the modality of diagnosis (microscopic confirmation or death certificate only) [39], [40], WACR data is superior to the European average published in EUROCARE-4 [46]. Microscopic confirmation is a particularly important indicator of data quality because histology is the ‘gold standard’ for cancer diagnosis and histological type is the primary basis for cancer classification. In our study, a very high proportion of cases were confirmed microscopically (>98%). The proportion of microscopically verified cases varied according to AYA category, and was lowest for CNS malignancies (88%). In the WACR database, <0.1% of tumour records were classified as DCO [41]. In our AYA series, DCO cases were rare (0.6% overall) with the exception of soft tissue sarcomas (0.3%).

Given the relative homogeneity of the Australian health system, and that WA is socio-demographically representative of Australia as a whole, these results may be considered to reflect AYA cancer outcomes nationally [42]. Clearly there are caveats on the applicability to other jurisdictions of our data on the relationship between geographical remoteness and outcomes, given that WA is characterised by singularly low population density and unusually long distances between rural/remote locations and urban specialist cancer facilities. The important issue of whether mean survival figures by geographical region are representative of wider areas as a whole has been addressed elsewhere [43], [44]. Additionally, as our data are derived from the relatively small WA population, outcomes of infrequent tumours, such as bone sarcomas, could not be properly examined. Hence survival probabilities and comparisons, especially for rare cancers, were susceptible to chance variation.

A major limitation of the present study was the lack of cancer staging data. We were unable to make a more in-depth assessment of differences in survival by stage at diagnosis because this information is not routinely collected by the WACR. Additional limitations include the potential for residual socio-economic confounding, as estimation of SES was based on the locality of residence at diagnosis, with measurement at the individual level not possible from de-identified linked data. Our approach may not have accurately captured some factors that contribute to cancer survival, such as healthy living environments, and adequate medical care. Additionally, AYAs are a heterogeneous group by virtue of transitioning through developmental life stages: some are dependent on parents and relatives while others provide for families of their own. As such, measuring SES as a single point-in-time geographical area composite variable may inadequately summarize an individual patient's life-course social and financial circumstances. On the other hand, only focusing on individuals ignores the broader issues of area contextual effects on health, such as community resources for healthy living.

An RSR estimate reflects the ratio of the observed survival divided by the expected survival of a cohort of the general population possessing similar characteristics with respect to age at calendar time/era and sex. Other major advantages of working with RSR estimates include the fact that data on cause of death are not required, which circumvents difficulties with inaccuracy or lack of death certification. However, this apparent strength may present important limitations for our AYA cohort. Young adults aged 20–39 years are the age group at the highest risk of acquiring HIV infection [45]; and infected individuals are at increased risk of developing Kaposi sarcoma, lymphoma and invasive cervical cancer, “acquired immunodeficiency syndrome (AIDS)-defining malignancies”, in this context. These cancers are more aggressive when occurring in HIV-infected patients, and result in poor prognosis. In our study, we did not ascertain HIV status and based our analysis on all-cause mortality without incorporating cause of death data, and were therefore unable to delineate between AIDS-related deaths and other deaths. Nonetheless, we do not expect AIDS-related deaths to have influenced our survival estimates. Likely due to WA’s geographical isolation, HIV infection is uncommon and the incidence of AIDS and related deaths is generally lower than in the rest of Australia and most industrialized countries. By mid-2007, only 475 diagnoses of AIDS and 324 deaths following AIDS were reported in WA since 1981 [45].

### Conclusion

Survival of AYAs diagnosed with cancer has generally increased over time. Despite favourable survival prognoses for some cancers in AYAs, there remains considerable disparity in cancer outcomes between different socio-demographic categories of AYA patients as well as substantial variation in the outcomes from different categories of cancer. Survival differentials identified in this study, particularly in relation to testicular germ cell tumours, should be investigated in greater depth; in order to distinguish instances in which improvements in survival can be attained through promoting equity of service access from those requiring novel therapeutic strategies directed towards distinctive aspects of tumour biology.
